# A case of an immunoglobulin G4 related inflammatory pseudotumor involving the clivus and the nasopharynx: A challenging case of a perplexing entity with an exhaustive overview

**DOI:** 10.1016/j.bas.2026.106134

**Published:** 2026-06-23

**Authors:** Mehdi Borni, Brahim Kammoun, Moncef Sellami, Saadia Makni, Chahir Kammoun, Issam Jardak, Noura Selem, Mohamed Zaher Boudawara

**Affiliations:** a Department of Neurosurgery, Habib Bourguiba University Hospital, Sfax, Tunisia; b Department of Otorhinolaryngology-Head and Neck Surgery, Habib Bourguiba University Hospital, Sfax, Tunisia; c Department of Pathology, Habib Bourguiba University Hospital, Sfax, Tunisia; d Private Practice Pathologist, Sfax, Tunisia; e Department of Nuclear Medicine, Habib Bourguiba University Hospital, Sfax, Tunisia

**Keywords:** IgG4-related disease, Inflammation, Clivus, MRI, Corticosteroids

## Abstract

**Introduction:**

Immunoglobulin G4-Related Disease (IgG4-RD) is a systemic fibroinflammatory condition characterized by IgG4-positive plasma cell infiltration and fibrosis, capable of affecting nearly any organ. While neurological manifestations are recognized, skull base and clival involvement are rare, and the spectrum of neurological presentations continues to expand.

**Research question:**

This case report addresses the diagnostic and therapeutic challenges of an exceedingly rare presentation of IgG4-RD involving the clivus and nasopharynx, focusing on its unique neurological phenotype and response to treatment.

**Material and methods:**

We report the case of a previously healthy 42-year-old male presenting with a three-year history of progressively intractable headaches. Initial evaluation included contrast-enhanced CT, MRI of the brain, cavum, and clival region, and a whole-body 18F-FDG PET-CT. Diagnosis was established via endoscopic biopsy of the nasopharyngeal/clival lesion followed by histopathological and immunohistochemical analysis. The patient was managed with systemic corticosteroid monotherapy.

**Results:**

Imaging showed an aggressive, hypermetabolic mass with clival osteolysis1. Histopathology confirmed IgG4-RD (IgG4+/total IgG + ratio >40\%, >100 IgG4+ cells/HPF). The patient presented with Arnold's neuralgia-like symptoms—a previously unreported neurological finding in IgG4-RD. Corticosteroid monotherapy resulted in a rapid, near-complete resolution of his headaches and favorable clinical evolution.

**Discussion and conclusion:**

This case highlights a unique, previously undescribed Arnold's neuralgia-like presentation of IgG4-RD involving the clivus and nasopharynx. Our experience supports the efficacy of corticosteroids as first-line treatment, even with rare anatomical involvement. This report underscores considering IgG4-RD in the differential diagnosis of atypical head and neck pain, particularly with clival lesions.

## Highlights

• **Patient Presentation:** A 42-year-old male presented with a three-year history of progressively intractable headaches. A unique and previously undescribed neurological manifestation was noted: **Arnold's neuralgia-like symptoms.**
• **Anatomical Involvement:** The disease involved a rare location: a mass in the **clivus and the nasopharynx.** Imaging showed an aggressive, hypermetabolic mass associated with **clival osteolysis.**
• **Diagnosis (Histopathology):** The diagnosis was confirmed histopathologically, showing dense lymphoplasmacytic infiltration with an **IgG4+/total IgG + plasma cell ratio exceeding 40%** and **>100 IgG4+ cells per high-power field (HPF).** Vaguely **storiform fibrosis** was also observed in the underlying stroma.
• **Treatment and Outcome:** The patient was treated with **systemic corticosteroid monotherapy.** This led to a **rapid and near-complete resolution** of his debilitating headaches and a **favorable clinical evolution**, demonstrating the efficacy of first-line treatment even in this rare location.

## 1 Introduction

IgG4-Related Disease (IgG4-RD) is a rare, immune-mediated condition first reported in Japan in 2001 and has since been increasingly recognized over the past two decades (Yamamoto et al., 2003) (Brito-Zerón et al., 2015). It encompasses a broad spectrum of previously unrelated, organ-specific diseases of unknown etiology, including Mikulicz disease, Küttner tumor (chronic sclerosing sialadenitis), Riedel thyroiditis, and autoimmune pancreatitis (Brito-Zerón et al., 2015).

IgG4-RD presents as an inflammatory disease that can affect any organ, associated with high levels of circulating IgG4, infiltration of IgG4-producing plasma cell complexes, and fibrosis of the affected organs (Yamamoto and Takahashi, 2014). It is, in fact, a systemic disease that usually affects multiple organs at the time of diagnosis. However, it can occasionally develop metachronously, affecting a single organ for many years, only to later involve others. At the skull and brain level, IgG4-RD can reach meninges, sphenoid and temporal bone, sphenoid and maxillary sinuses, clivus, blood vessels, trigeminal nerve, brain parenchyma, infratemporal fossa, pterygopalatine fossa, and suprasellar region (Cler et al., 2021). The clivus involvement remains very rare and only few cases have been reported in the literature (Cler et al., 2021). The disease shows a male predominance (male-to-female ratio of approximately 3:2) and primarily affects individuals over 60 years of age (Brito-Zerón et al., 2015), although several pediatric cases have also been reported (Nambirajan et al., 2019) (Sane et al., 2013).

Differential diagnosis of IgG4-RD lesions is critical. These lesions must be carefully distinguished from benign neoplasms such as hemangiomas and meningiomas, as well as malignant conditions including carcinomas and lymphomas. Neurological manifestations of IgG4-RD can mimic those of tumors, infections, and other inflammatory disorders, making accurate diagnosis particularly challenging (Chen et al., 2023).

Despite its rarity and the current lack of comprehensive epidemiological data, we report a newly identified case of IgG4-RD involving the clivus and the nasopharynx in a 42-year-old male patient who presented with intractable headache. The patient demonstrated a favorable clinical response to immunosuppressive therapy. In this report, we review the relevant literature to discuss the clinical presentation, diagnostic approach, therapeutic strategies, and prognosis of this uncommon inflammatory disorder.

## 2 Case report

A previously healthy 42-year-old male, with no significant medical or surgical history (non-smoker), presented with a three-year trajectory of progressively intractable headaches. The patient, an engineer and father of two healthy offspring, initially characterized the cephalgia as throbbing and biparietal in origin, subsequently evolving to a holocranial distribution. These episodes predominantly manifested in the morning, with reported nocturnal exacerbations. Concomitant symptoms included recurrent episodes of morning nausea without emesis. Initially, the headaches demonstrated transient responsiveness to conventional first-line analgesics; however, their refractoriness to treatment incrementally increased over time. The headaches were associated with acute cervical and occipital pain radiating to the frontal region and orbits, accompanied by paresthesia in the nuchal and cranial integuments, suggestive of an Arnold's neuralgia-like presentation. Notably, the patient reported the absence of concomitant visual disturbances, convulsive episodes, or other focal neurological deficits throughout the clinical course. Furthermore, he remained afebrile. Comprehensive clinical examination, including funduscopic evaluation, yielded no discernible abnormalities, specifically precluding findings consistent with papilledema. The presentation was additionally complicated by heightened scalp allodynia.

A contrast-enhanced computed tomography (CT) of the brain (Fig. 1) revealed a slightly hyperdense tumor mass involving the roof, as well as the posterior and posterolateral walls of the right side of the nasopharyngeal cavity. The lesion filled the Rosenmüller fossa and obstructs the right tubal orifice. Anteriorly, the mass protruded into the area of both choanae. Posteriorly, it extended into the long muscles of the neck and is associated with invasion and osteolysis of the clivus and the right occipital condyle. These findings were suggestive of an aggressive lesion with significant local infiltration of both soft tissue and bony structures. To better explore this lesion, a magnetic Resonance Imaging (MRI) of the brain, cavum, and clival region (Fig. 2, Fig. 3) was performed and revealed the tumor mass located in the roof and posterior as well as posterolateral walls of the right side of the nasopharyngeal cavity. The lesion occupied the Rosenmüller fossa and obstructed the right-sided tubal orifice. On imaging, the mass appeared hypointense on T1-weighted sequences, demonstrated intermediate signal intensity on T2-weighted sequences, with fairly homogeneous enhancement after gadolinium injection. This lesion measured approximately 50 × 28 mm in the axial plane and 29 mm in craniocaudal height. Anteriorly, it protruded into both choanae, while posteriorly it extended into the longus capitis and longus colli muscles. It caused notable osteolysis and invasion of the clivus and the right occipital condyle. Laterally, the mass infiltrated the right parapharyngeal fat and extended into both the pre- and retro-styloid compartments of the parapharyngeal space. Superiorly, it eroded the inferior wall of the sphenoid sinus. Inferiorly, there was no involvement of the oropharynx, hypopharynx, or larynx. No evidence of endocranial extension was present. There was no cervical lymphadenopathy. However, partial opacification of the right mastoid air cells was observed. Cervical MRI showed no abnormalities apart from cervical straightness with a tendency towards inversion of cervical lordosis. The thoracoabdomino-pelvic CT scan (Fig. 4) did not reveal any other abnormalities.

**Fig. 1:**
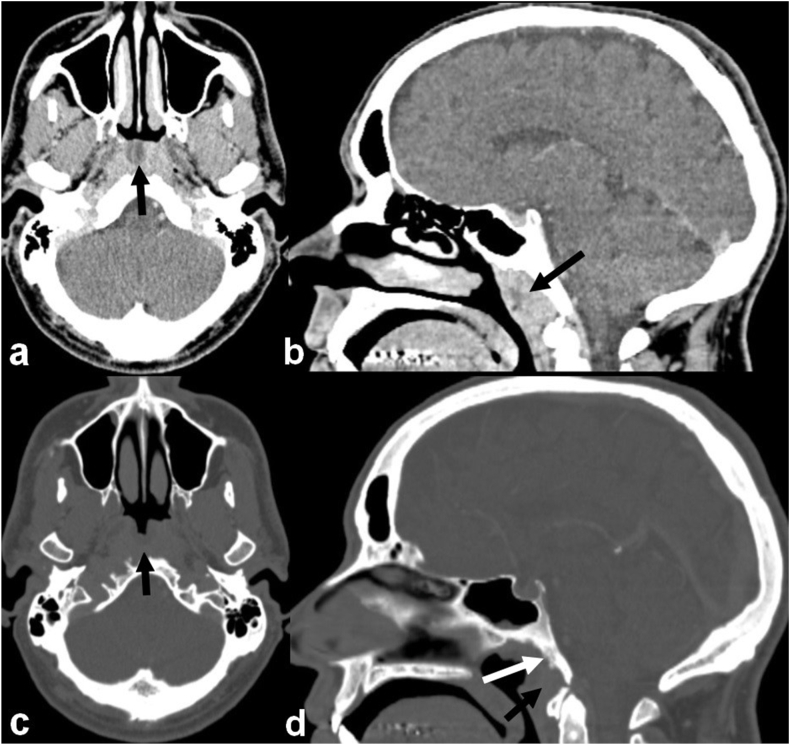
Axial (**a**, **c**) and sagittal (**b**, **d**) contrast-enhanced computed tomography (CT) of the brain, displayed in parenchymal (**a**, **b**) and bone (**c**, d) windows. The scans reveal a slightly hyperdense tumor mass (black arrows) involving the roof, posterior, and posterolateral walls of the right nasopharyngeal cavity. The lesion is associated with invasion and osteolysis of the clivus and the right occipital condyle (**d**, white arrow).

**Fig. 2:**
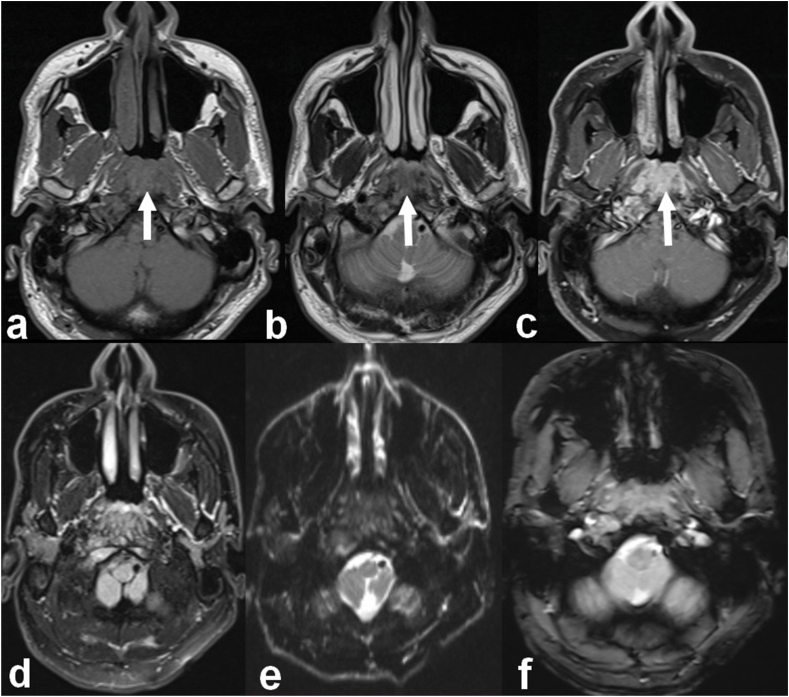
Axial T1-weighted magnetic resonance imaging (MRI) demonstrates a lesion located in the roof and posterior as well as posterolateral walls of the right side of the nasopharyngeal cavity and the clivus exhibiting hypointense signal characteristics (**a**, white arrow). On axial T2-weighted MRI, the lesion displays intermediate signal intensity (**b**, white arrow). Following intravenous administration of a gadolinium-based contrast agent, axial T1-weighted MRI reveals fairly homogeneous enhancement of the lesion (**c**, white arrow). The lesion measures approximately 50 mm in its maximal axial dimension and 28 mm in the orthogonal axial dimension, with a craniocaudal extent of 29 mm. There is no significant associated vasogenic edema identified on the axial Fluid-Attenuated Inversion Recovery (FLAIR) sequence (**d**). Axial Diffusion-Weighted Imaging (DWI) does not reveal restricted diffusion, as evidenced by the absence of hyperintensity (**e**). Axial Gradient Echo (GRE) sequence does not demonstrate evidence of calcification or intratumoral hemorrhage, as indicated by the absence of susceptibility artifact (**f**).

**Fig. 3:**
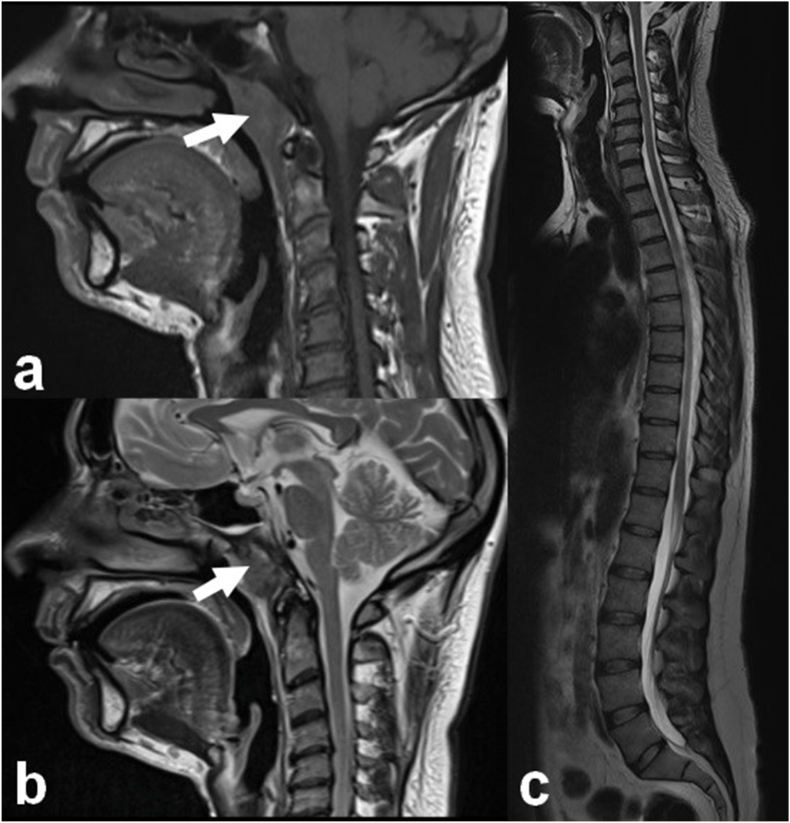
Sagittal T1-weighted magnetic resonance imaging (MRI) demonstrates a tumor located at the level of the nasopharyngeal cavity and the clivus, exhibiting hypointense signal characteristics (**a**, white arrow). Sagittal T2-weighted MRI reveals the tumor displaying intermediate signal intensity (**b**, white arrow). A full spine MRI on T2-weighted sequences does not reveal any significant abnormalities (**c**, white arrow).

**Fig. 4:**
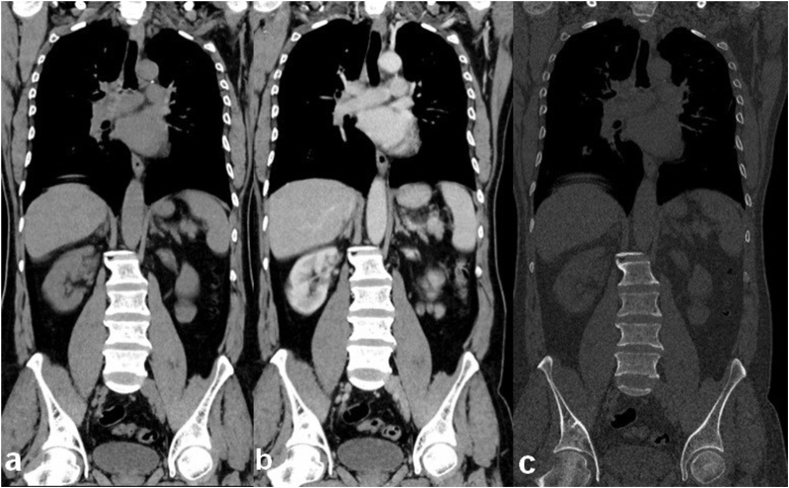
Coronal thoracoabdomino-pelvic CT scan: Pre-Contrast (**a**), venous phase (**b**), and bone window (**c**) - No significant abnormalities detected.

To search for other distant tumor locations, a whole-body 18F-Fluorodeoxyglucose Positron Emission Tomography-computed Tomography scan (18F-FDG PET-CT) (Fig. 5) was performed following intravenous administration of 8.35 mCi of fluorodeoxyglucose, corresponding to an effective dose of 6.51 mSv. The patient's blood glucose level was 1.07 g/L at the time of injection. Image acquisition commenced 1 h post-injection, beginning with a skull base CT scan to mid-thigh (120 kV, 100 mAs; CTDI: 9.60 mGy; DLP: 973 mGy·cm), followed immediately by PET imaging. At the cervical level, there was intense hypermetabolic activity (SUVmax: 13.9) corresponding to a soft tissue mass involving the roof, posterior, and posterolateral walls of the right nasopharynx. The lesion extended superiorly and posteriorly to the skull base. Additionally, mildly hypermetabolic bilateral jugulo-carotid lymph nodes were observed, with a right-sided SUVmax of 1.63 at the first hour, increasing to 3.35 on delayed imaging at the second hour, suggestive of possible reactive or low-grade metastatic involvement. A moderate oropharyngeal and laryngeal hypermetabolism, likely of inflammatory origin, was also noted. In the thoracic region, there was no evidence of pathological FDG uptake in the pulmonary parenchyma or pleura, and no hypermetabolic activity was observed in the mediastinal, hilar, or axillary lymph nodes. In the abdominopelvic region, normal physiological FDG uptake was noted in the liver, spleen, pancreas, kidneys, and adrenal glands. There was no abnormal uptake in the stomach or any lymphadenopathy with pathological FDG activity in the abdominal or pelvic regions. All these aspects were in favor of a metabolically active nasopharyngeal tissue mass. Bone window analysis showed no evidence of pathological skeletal hypermetabolism throughout the scanned area. These feature suggested a metabolically active nasopharyngeal lesion.

**Fig. 5:**
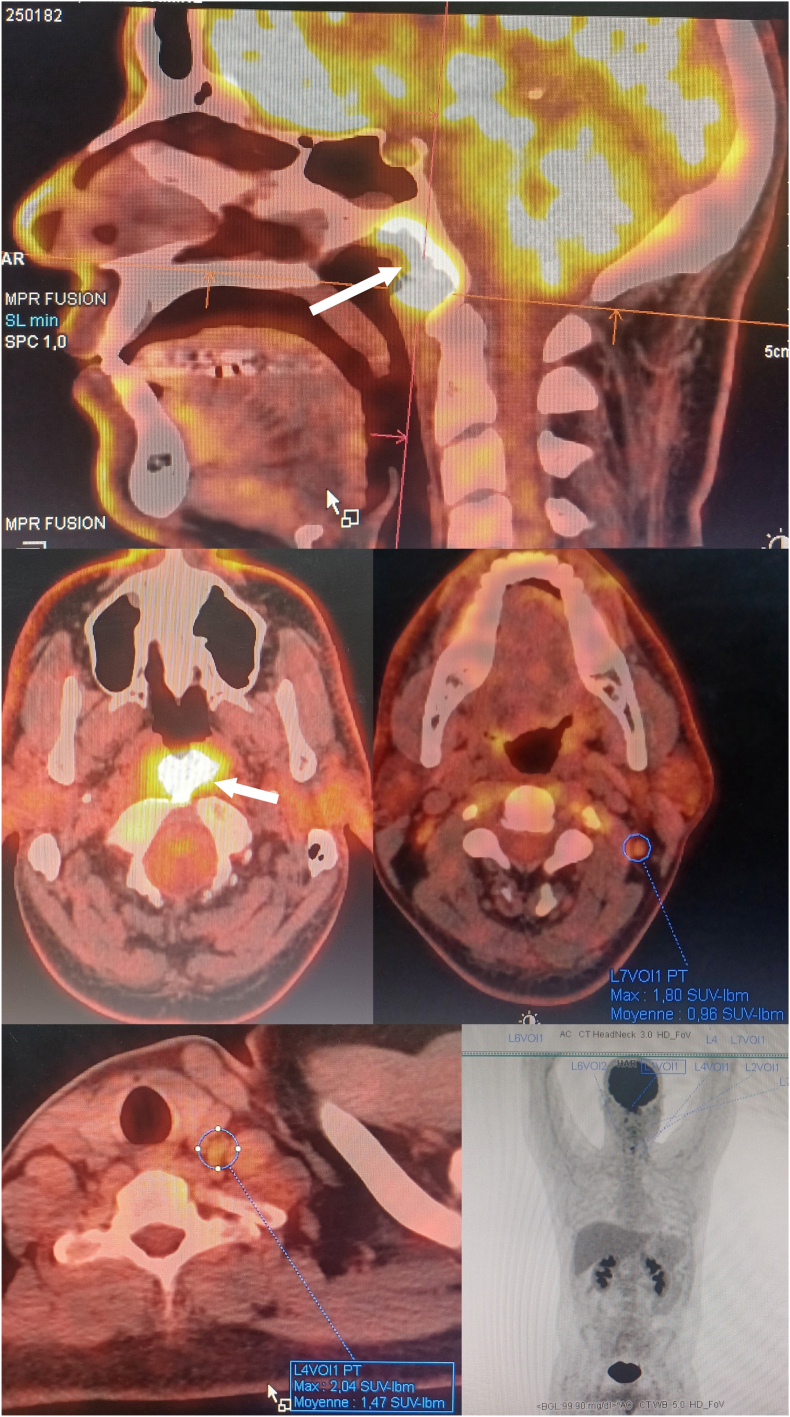
Sagittal, axial and coronal 18F-Fluorodeoxyglucose Positron Emission Tomography-computed Tomography scan (18F-FDG PET-CT) images showing intense hypermetabolic activity (SUVmax: 13.9) (white arrows) corresponding to a soft tissue mass involving the roof, posterior, and posterolateral walls of the right nasopharynx, with superior and posterior extension to the skull base. Mildly hypermetabolic bilateral jugulo-carotid lymph nodes are observed (SUVmax: right 1.63 at first hour, increasing to 3.35 on delayed imaging), suggestive of possible reactive or low-grade metastatic involvement. Additional moderate oropharyngeal and laryngeal uptake likely reflects inflammatory changes. No pathological FDG uptake is seen in thoracic, abdominal, or skeletal regions.

The patient underwent an endoscopic biopsy of the nasopharyngeal and clival submucosal lesion (Fig. 6). The procedure was conducted under general anesthesia to ensure patient comfort and immobility. A 4.0 mm diameter, 30-degree rigid endoscope was meticulously utilized to facilitate precise visualization and targeted procurement of tissue fragments from the identified lesion. This approach allowed for minimally invasive access to the anatomically complex region and the acquisition of representative histological material for subsequent pathological evaluation. The postoperative period was uneventful, indicating an absence of immediate complications. All resected tissue samples, meticulously retrieved during the surgical procedure, were promptly fixed in a 10% neutral buffered formaldehyde solution. This crucial step was performed to preserve cellular morphology and prevent autolysis, ensuring the integrity of the specimens for subsequent histopathological analysis at the specialized pathology laboratory.

**Fig. 6:**
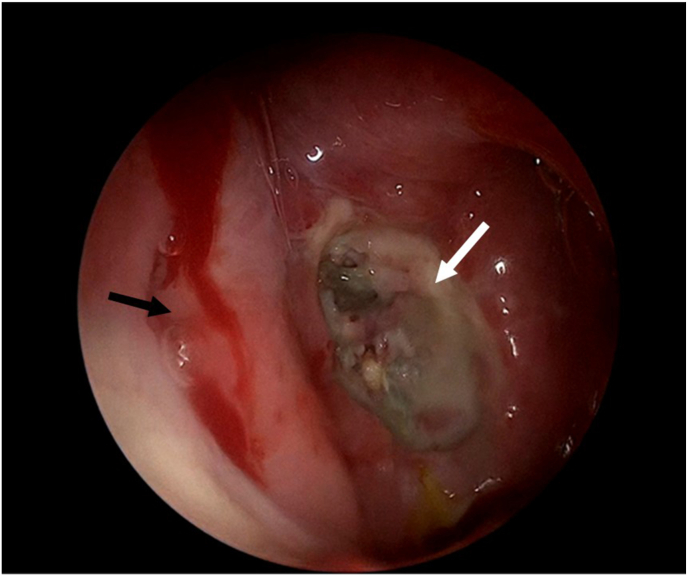
Intraoperative nasal endoscopy via the right nasal fossa revealed an ulcerated lesion on the posterior wall of the nasopharynx (white arrow), characterized by granulomatous content. The pharyngeal opening of the Eustachian tube (black arrow) was also visualized.

The histopathological examination (Fig. 7) revealed mucosa lined by a non-atypical, regular respiratory epithelium. The underlying stroma demonstrates a dense and polymorphic inflammatory infiltrate, prominently featuring hyperplastic lymphoid follicles with preserved germinal centers. The interfollicular areas were notably enriched in plasma cells. In addition, the stroma showed areas of diffuse fibrosis with a vaguely nodular architecture. Importantly, no evidence of obliterative phlebitis is identified. There were no granulomatous formations suggestive of a tuberculoid etiology, and no histological features indicative of carcinomatous proliferation were observed. An immunohistochemical panel was performed, including antibodies against cytokeratin, CD3, CD20, anaplastic lymphoma kinase (ALK), and immunoglobulin G4 (IgG4). The lesion was negative for cytokeratin and ALK, effectively ruling out epithelial and ALK-positive lymphoproliferative neoplasms. CD3 and CD20 immunostaining highlighted a mixed population of reactive T and B lymphocytes, respectively, consistent with a polyclonal immune response. IgG4 immunostaining revealed strong but heterogeneous positivity, with IgG4-positive plasma cells accounting for approximately 40% of the total inflammatory infiltrate and exceeding 100 IgG4-positive cells per high-power field (HPF), raising consideration for an IgG4-related disease process.

**Fig. 7:**
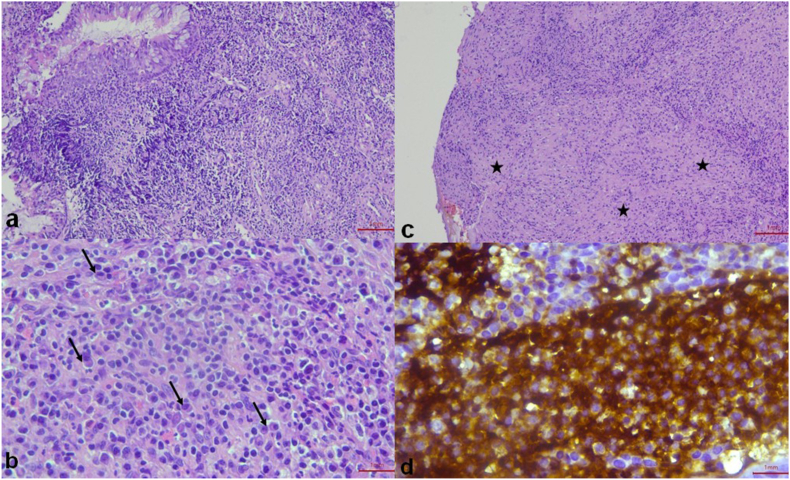
Photomicrograph of the histological examination showing a dense lymphoplasmacytic inflammatory infiltrate within the chorion [**a**, hematoxylin and eosin (H&E), 100x magnification], indicating a significant immune response. Notably, the underlying stroma exhibits vaguely storiform fibrosis (**b**, H&E, 100x magnification, asterisk), a pattern characterized by collagen fibers arranged in a swirling or matted configuration, often seen in fibroinflammatory conditions. At higher magnification (**c**, H&E, 400x magnification), the inflammatory infiltrate is remarkably rich in plasma cells (black arrows), suggesting a prominent humoral immune component. Crucially, immunohistochemical staining for IgG4 (**d**, IgG4, 400x magnification) demonstrates a strikingly elevated number of IgG4-positive plasma cells, exceeding 100 cells per high-power field (HPF).

The therapeutic approach initiated involved the administration of a standardized regimen of intravenous corticosteroid boluses. This powerful class of anti-inflammatory agents is a frequently employed and reliable intervention for rapidly mitigating acute inflammatory processes believed to underpin a variety of neurologically mediated symptom complexes, particularly when a suspected mechanism is autoimmune or inflammatory. The patient's response to this aggressive treatment strategy was both pronounced and remarkably rapid, serving as a strong ex juvantibus confirmation of an inflammatory etiology. Specifically, the patient reported a near-complete resolution of the previously intractable, three-year history of debilitating headaches by the time the treatment course was completed, confirming the efficacy of the intervention in arresting the underlying pathological cascade. This significant positive response strongly suggested a corticosteroid-responsive etiology for the patient's symptoms. Consequently, given the favorable immediate outcome and the need for comprehensive systemic evaluation, the patient was discharged with a plan for continued outpatient follow-up and formally referred to the internal medicine department for further diagnostic elucidation and the establishment of an appropriate long-term management strategy.

## 3 Discussion

IgG4-related disease is a fibroinflammatory disorder characterized by several clinical manifestations of systemic predominance. The main organs involved are the salivary glands, bile ducts, pancreas, retroperitoneum, lungs, trachea, and skin. Certain diseases that were previously classified as distinct entities with their respective eponyms, such as Mikulicz disease (salivary and lacrimal gland involvement), Küttner tumor (isolated submandibular gland involvement), autoimmune pancreatitis, and sclerosing cholangitis, are now recognized as part of the IgG4-related disease spectrum (Stone et al., 2013) (Takahashi et al., 2010). The key feature of the immune response in IgG4-related disease (IgG4-RD) is the infiltration of CD4^+^ cytotoxic T lymphocytes (CTLs), which are activated by B cells. The formation of inflammatory masses composed of immune cells and fibrotic tissue in IgG4-RD likely involves a coordinated interplay between macrophages, activated B cells, CD4^+^ CTLs, and fibroblasts (Perugino, 2020). Friedrich et al. (2021) investigated the interactions between T cells, focusing on the chemokine CCL5—expressed by CD8^+^ effector memory T cells—which binds to CCR4/5 receptors on other T cells. Their findings suggest that this T-cell crosstalk facilitates the recruitment of both helper and cytotoxic CD4^+^ T cells, which are implicated in driving the chronic inflammatory response observed in IgG4-RD (Friedrich et al., 2021). In our patient, CD3 and CD20 immunostaining highlighted a mixed population of reactive T and B lymphocytes, respectively, consistent with a polyclonal immune response.

In healthy individuals, IgG4 constitutes a minimum of 5% of the total IgG antibody population (Takahashi et al., 2010). Importantly, immunological investigations have demonstrated that IgG4 does not trigger the classical complement pathway and has historically been thought to have a restricted function in immunological responses (Cler et al., 2021). A notable feature of roughly 50% of IgG4 molecules is the weak, non-covalent linkage between their heavy chains. This loose association allows these chains to dissociate and reassemble in different pairings. As a consequence, individual IgG4 antibodies can bind to distinct sites on antigens, which ultimately inhibits their capacity to form immune complexes (Aalberse, 2002).

In 2019, the American College of Rheumatology established diagnostic criteria for IgG4-related disease (IgG4-RD), based on a combination of characteristic clinical features and the exclusion of alternative diagnoses (Wallace et al., 2020). The hallmark histopathological findings include dense lymphoplasmacytic infiltration, storiform fibrosis, and obliterative phlebitis. A diagnosis of IgG4-RD is supported when the ratio of IgG4^+^ to total IgG^+^ plasma cells exceeds 40%, and there are more than 10 IgG4^+^ plasma cells per high-power field (HPF) (Wallace et al., 2020). In our present case, the diagnosis of an IgG4-related inflammatory disease was made based on histopathological evidence, including granulation tissue with numerous new blood vessels with swollen endothelial cells, fibrosis and a prominent mixed inflammatory infiltrate composed of hyperplasic lymphocytes which contained several follicles exhibiting clear germinal centers, plasma cells, and polymorphonuclear cells. Additionally, foci of ischemic necrosis were observed. IgG4 immunostaining revealed strong but heterogeneous positivity, with IgG4-positive plasma cells accounting for approximately 40% of the total inflammatory infiltrate and exceeding 100 IgG4-positive cells per high-power field.

The epidemiology of IgG4-related disease has not been fully established. Most cases have been described in Japan (Aalberse, 2002). One study showed an estimated incidence of 1.08 cases per 100,000 population in 2009 and a prevalence of 0.006% for the same year, with a mean age of 60 years at diagnosis and a male predominance like our patient. However, diagnosis is difficult due to a lack of familiarity with IgG4-related disease, so this prevalence is poorly estimated (Uchida et al., 2012).

Generally, IgG4-RD can affect the entire human body. The nervous system may also be affected, mainly the skull and meninges according to some recent studies (Deshpande, 2015) (Toyoda et al., 2012). A systematic literature review carried out by Cler et al. (2021) in 2021, following PRISMA guidelines, revealed 184 cases of IgG4-RD in the skull base or calvarium across 113 identified reports. The most commonly involved anatomical sites were the meninges, posterior fossa, cavernous sinus, clivus, and mastoid bone. Among these case, clivus was involved in only 11cases (Cler et al., 2021). More recently, and through a more exhaustive study of the literature, we were able to identify 7 other articles (Liu et al., 2021; Suzuki et al., 2021; Kaneko et al., 2024; Yamamuro et al., 2021; Liu et al., 2022; Sahoo et al., 2023; Coviello and Locke, 2022) reporting IgG4-related clival disease between 2021 and 2024 to total a number of 18 cases over a period of 10 years from 2014 to 2024. To the best of our knowledge, our patient would be the 19th case in the literature reporting this type of rare pseudotumoral inflammatory pathology with an anatomical location that is also quite rare.

According to the previous reports, magnetic resonance imaging (MRI) of IgG4-related lesions typically demonstrates iso-intense signals on T1-weighted images and low to iso-intense signals on T2-weighted images. Following gadolinium administration, the lesions generally exhibit marked contrast enhancement (Tang et al., 2018). These radiological characteristics are consistent with findings from a systematic literature review published in 2015 by Spinazzi et al. (2015), which reported that 75% of IgG4-related lesions appeared isointense on T1-weighted MRI, while 62.5% showed hypointensity on T2-weighted MRI. Additionally, all reviewed cases demonstrated positive enhancement following contrast administration. In our patient, the same features were observed. Conversely, computed tomography (CT) studies, while exhibiting non-specific radiological features for immunoglobulin G4-RD, may demonstrate lesions with increased thickness and hyperattenuation as it was seen in our case. These lesions typically exhibit contrast enhancement following the administration of iodinated contrast media.

Metabolically speaking, the 18F-FDG PET-CT is now a valuable tool for assessing IgG4-RD, particularly in identifying multi-organ involvement and guiding biopsy. It may reveals increased metabolic activity in affected tissues, as it was seen in our patient. PET/CT is also useful for monitoring response to corticosteroids, as metabolic activity tends to decrease with successful treatment. However, due to the lack of specificity, its findings should be interpreted in conjunction with clinical, serological, and pathological data to distinguish IgG4-RD from other malignancies or infections (Zhang et al., 2014; Ebbo et al., 2017). As for investigating the skull base lesion, the usefulness of PET/CT is questionable. In this topic, Hines et al. (2016) published in 2016 a study and found that while it's a valuable tool, its positive predictive value for inflammation varies depending on the pre-test probability and the potential for false positives from malignancy or benign metabolically active lesions. Conversely, the negative predictive value tends to be higher, making a negative PET-CT more reliable for excluding malignancy, though false negatives can occur. The authors' case series and systematic literature review likely highlighted the heterogeneity of skull base lesions and the need for careful interpretation of PET-CT findings in conjunction with clinical and other imaging data. In our patient, we performed PET/CT in conjunction with brain CT and MRI to help support the diagnosis and not to retain IGG4-RD since the diagnosis was ultimately made only by histology.

The differential diagnosis of IgG4-RD affecting the brain encompasses a spectrum of inflammatory, autoimmune, infectious, and neoplastic conditions. IgG4-related hypertrophic pachymeningitis must be distinguished from other causes of dural thickening like neurosyphilis, tuberculosis, sarcoidosis, and ANCA-associated vasculitides. Similarly, IgG4-related hypophysitis can mimic other forms of pituitary inflammation, tumors such as adenomas and meningiomas, and infections. Brain parenchymal lesions in IgG4-RD can resemble neurosarcoidosis, lymphoma, or metastatic disease (Chen et al., 2023) (Deshpande et al., 2012) (Baptista et al., 2017).

Clinically, and consistent with the systematic literature review by Cler et al. (2021), the spectrum of clinical manifestations ranges from asymptomatic incidental findings to a variety of neurological symptoms. These include cephalalgia as it was seen in our patient, visual impairment, retro-orbital pain, auditory dysfunction (encompassing hearing loss and other otologic symptoms), intracranial hypertension, vasculopathy, facial pain, seizures, hemiparesis, cranial nerve deficits, and other generalized neurological signs and symptoms. Arnold's neuralgic presentation was not seen in IGG-4 disease and our patient would be the first to report this type of neuropathy. Our case appears to be the first documented instance of such a specific neuropathic presentation in the spectrum of IgG4-related disorders, thereby expanding the known phenotypic diversity of this multifaceted disease and warranting further investigation into the precise mechanisms underlying this neurological involvement.

There is little evidence to support treatment options for IgG4-RD. Nevertheless, it is well recognized that when vital organs are affected, more aggressive therapeutic interventions are warranted. In the absence of appropriate treatment, the disease may progress to irreversible fibrosis (Stone et al., 2013). The primary treatment for IgG4-related disease (IgG4-RD) to date has been corticosteroids, with a tapering course of prednisone typically producing a rapid clinical response. This responsiveness is often considered a supportive diagnostic feature of IgG4-RD (Wallace et al., 2020). In cases of systemic involvement, glucocorticoid therapy may be associated with an increased risk of relapse; however, this trend has not been clearly established for lesions involving the skull base and calvarium (Khosroshahi, 2011). A multicenter phase II prospective clinical trial reported in 2016 by Masaki et al. (2017) which evaluated the effectiveness of glucocorticoid (prednisolone at 0.6 mg/kg tapered over time) for previously untreated patients with definite IgG4-RD. The primary outcome was the complete remission rate at one year. Out of 61 enrolled patients, 44 met the criteria for definite IgG4-RD. The study found that 65.9% of these patients achieved CR, and the overall response rate was 93.2%, with no patients being resistant to the initial steroid treatment. The most common side effect was glucose intolerance, and the relapse rate was 6 out of 44 patients. The authors concluded that glucocorticoids are generally effective for IgG4-RD and highlighted the importance of accurate diagnosis, as some patients were initially misdiagnosed.

Adjuvant immunosuppressive agents, such as azathioprine, mycophenolate mofetil, or rituximab, have also been utilized. Over time, treatment protocols have increasingly favored the combination of corticosteroids with rituximab rather than other immunosuppressive regimens. Rituximab, in particular, has demonstrated superior efficacy in systemic IgG4-RD and is recommended for patients with severe or recurrent disease, or in those who are unable to tolerate corticosteroids. In refractory cases, intrathecal administration of rituximab has been explored and has shown success (Della-Torre et al., 2018) (Soares et al., 2019). In cases of steroid-refractory disease, local radiation therapy has demonstrated efficacy in a subset of patients (Deshpande et al., 2012). It is important to remember that, due to the risk of corticosteroids and immunosuppressive agents exacerbating mimicking conditions like osteomyelitis or interfering with biopsy results, these therapies should be deferred until a high clinical suspicion for IgG4-related disease (IgG4-RD) exists (Önder et al., 2015). In this particular patient, our treatment regimen was exclusively based on the administration of systemic corticosteroids. We deliberately chose not to introduce adjuvant immunosuppressive agents, a decision that ultimately correlated with a favorable clinical evolution. This outcome suggests that for this specific case, corticosteroid monotherapy was sufficient to achieve a positive response, circumventing the potential side effects associated with broader immunosuppression.

Regarding the prognosis, IgG4-RD has generally favorable one, particularly when diagnosed and treated promptly. However, its clinical course is variable: while some patients may experience spontaneous remission, others follow a relapsing-remitting pattern with potentially increased mortality risk. This heightened risk is associated with extensive organ involvement—especially of the kidneys, pancreas, aorta, and liver—multi-organ disease, renal impairment, the potential development of malignancies, and complications related to immunosuppressive therapy (Wallace et al., 2024). Therefore, careful monitoring for disease relapse, ongoing assessment of organ function, and proactive management of treatment-related side effects are critical to improving long-term outcomes in affected patients (Katz, 2022; Duggal et al., 2021).

Despite growing awareness, IgG4-related disease involving the skull base—including the clivus—remains a relatively rare condition, and optimal management strategies continue to evolve. To advance clinical care, there is a critical need for prospective, multi-center studies with larger patient populations. Such research is essential to develop standardized treatment protocols, identify reliable biomarkers for disease activity and prognosis, and ultimately enhance long-term outcomes for individuals affected by this complex disorder.

## 4 Strengths and limitations

The work's value lies in its documentation of an extremely rare case of IgG4-RD involving the clivus and nasopharynx. Its primary strength is the identification of a novel neurological presentation—Arnold's neuralgia-like symptoms—which expands the known phenotypic spectrum of the disease. The report further reinforces the therapeutic strategy by demonstrating a rapid and favorable response to corticosteroid monotherapy, even with aggressive-looking involvement. The main limitation is that, as a single case report, its unique findings are not generalizable, and it lacks the long-term follow-up data necessary to assess the risk of relapse or long-term prognosis, which is critical in managing this systemic disease.

## 5 Conclusion

IgG4-related disease, a systemic fibroinflammatory pathology of incompletely elucidated pathogenesis, exhibits a recognized capacity for CNS involvement, occurring either as an isolated manifestation or in conjunction with multi-organ involvement. Its propensity to present as mass-forming lesions frequently leads to its misidentification as neoplastic processes. Consequently, a high index of suspicion for IgG4-RD is critical in the differential diagnosis. Accurate diagnosis, informed by the lesion's immunophenotypic profile, integrated with radiological findings and elevated serum IgG4 concentrations, is essential for initiating appropriate immunosuppressive therapy. This entity holds significant relevance in contemporary neuropathology due to its inflammatory etiology and the typically robust therapeutic response to corticosteroids, resulting in a generally favorable prognosis.

## Conflicts of interest

The authors declare that there are no conflicts of interest including financial, consultant, institutional and other relationships that might lead to bias or to a conflict of interest.
